# Residency as a determinant of oral health disparities in primary dentition among HIV-infected, HIV-exposed, and unexposed children in Kenya – findings from a cohort study

**DOI:** 10.1080/09581596.2025.2595527

**Published:** 2025-12-01

**Authors:** Ana Lucia Seminario, Sydney Malone, Immaculate Opondo, Whasun Oh Chung, Arthur Kemoli, Yan Wang

**Affiliations:** a Pediatric Dentistry, University of Washington, Seattle, Washington, USA;; b Pediatric Dentistry, Maseno University, Kenya;; c Oral Health Sciences, University of Washington, Seattle, Washington, USA;; d Pediatric Dentistry, University of Nairobi, Nairobi, Kenya;; e School of Dentistry, University of California Los Angeles, Los Angeles, California, USA

**Keywords:** HIV, children, oral health, residency, primary dentition

## Abstract

Residency is a critical factor related to the social determinants of health. This study aimed to evaluate the role of residency in oral health disparities among Kenyan children during the primary dentition period. We recruited participants from three cohorts of children, all aged 3 years, including those living with HIV infection (HIV), exposed to HIV but uninfected (HEU), and their healthy peers (HUU; uninfected and unexposed). We recruited 120 children in each cohort. Logistic regression was used to estimate the associations between oral diseases, HIV status, and residence, adjusting for sex and school type. Most children (47%) were enrolled in private schools, and 49% lived in rural areas. Children in HIV cohort had a significantly higher prevalence (81%) of abnormal oral findings compared with those in HEU (60%) and HUU (54%) cohorts (p < 0.0001). Children living in rural areas (OR = 3.3, 95% CI:1.7–6.3) and urban areas (OR = 2.0, 95% CI:1.1–3.9) were more likely to have abnormal findings than those in peri-urban areas. However, peri-urban residents had the highest prevalence of dental caries (63%), while rural residents had the highest combined prevalence of all dental conditions (83%). Residential area contributed to differences in the burden of oral diseases among HIV and HEU cohorts. Targeted prevention and treatment strategies should be considered by residential location.

## Introduction

Even though human immunodeficiency virus (HIV) has become a controllable chronic condition with proper care (Sematlane et al., 2021) significant disparities persist in Sub-Saharan Africa regarding awareness, treatment, and viral suppression (Dwyer-Lindgren et al., 2019; Moyo et al., 2023). For decades, an arbitrary age of 15 years has been used to define pediatric (0–14 years) and adult (15 and above) populations in HIV programs and data collection (Rakhmanina et al., 2024). According to the latest data, an estimated 120,000 children aged 0–14 acquired HIV worldwide in 2023, with most from Africa (65,000) (The Foundation for AIDS Research (amfAR), 2023). Despite comprising only 3% of the global HIV population, children in this age group accounted for 12% of AIDS-related deaths (76,000 children) in 2023 (Payagala & Pozniak, 2024).

Children who are HIV-exposed uninfected (HEU) are those exposed to HIV in utero and during breastfeeding but remain uninfected (Slogrove et al., 2020). The HEU population is growing rapidly, with nearly one million infants born each year (Wedderburn et al., 2019). Literature suggests that HEU children, particularly in low- and middle-income countries, are at risk for poor health outcomes (Wedderburn et al., 2019). However, research on this population remains limited, especially regarding oral health. Interpreting studies on HEU children presents challenges due to differences in exposure between high- and low-income settings (Wedderburn et al., 2019; 2023). These differences include the duration of HIV exposure (prenatal and postnatal), ART exposure, and variations in antiretroviral drug classes and combinations (Wedderburn et al., 2019). HEU children experience higher morbidity and mortality than HIV-unexposed uninfected (HUU) children during the first 1–2 years of life (Arikawa et al., 2016; Labuda et al., 2020). A meta-analysis conducted in 2016, which included 22 studies, found that HEU children have a 60–70% higher mortality risk compared to HUU children across all age groups (<1 year, 1–2 years, and >2 years) (Brennan et al., 2016).

Children in HIV cohort (CHIV) have a higher prevalence of hard and soft tissue diseases due to their compromised immune system (Ramos-Gomez & Folayan, 2013) and oral diseases are often neglected (Lam et al., 2022; Lauritano et al., 2020; Ramos-Gomez & Folayan, 2013; Wang et al., 2023). Thus, studies on oral health among CHIV or HEU children remain limited. One study indicated that caries experience among children aged 5–7 years does not differ significantly between HEU and HUU children (Birungi et al., 2020). Another study found that HIV-infected children aged 6 to 72 months had higher odds of developing caries compared to HUU children. However, no significant difference was observed between HEU and HUU children (Coker et al., 2018). In Kenya, caregivers reported 42% of CHIV children, 27% of HEU children, and 17% of HUU children had at least one oral disease being oral candidiasis was the most reported condition (Seminario et al., 2021). Yet, data on oral health outcomes in children aged 3–5 years remained unknown.

Where one lives can impact an individual’s health. There are significant health disparities in HIV infection rates between urban and rural areas (Magadi, 2017). The epicenter of HIV is beginning to shift toward suburban and rural areas in the U.S. and Canada (Schafer et al., 2017). The urban-rural gap impacts HIV transmission and viral load testing in Eastern African countries (Maulide Cane et al., 2021; Parker et al., 2021). Social cohesion tends to be lower in urban than in rural communities, and the urban poor are particularly vulnerable (Magadi, 2017). Urbanization patterns and increasing urban poverty also influence the HIV epidemic and contribute to oral health disparities in Kenya. The most recent Kenya Population-Based HIV Impact Assessment (KENPHIA), conducted in 2018, estimated that 140,000 CHIV in the country, yet only 49% achieved viral suppression (Mutisya et al., 2022). The latest AIDS Strategic Framework of Kenya identified 13 counties as priority based on their increasing incidence when compared to overall prevalence (National AIDS Control Council, 2020). This national report prioritized HIV resources for high-risk counties including Kisumu County (West Kenya), which ranked third in highest incidence of new infections in Kenya (National AIDS Control Council, 2020). The 2019 estimate of the population of children aged 0–14 who are HEU in Kenya was 840,000 (650,000–1,100,000), and the number continued to grow (Slogrove et al., 2020).

The goal of our study was to understand the impact of HIV infection and exposure on oral health among 3- to 4-year-old children in Western Kenya. We aimed to evaluate how HIV exposure status influences oral disease prevalence in this population. We hypothesized that CHIV children have a significantly higher prevalence of oral diseases compared to their HEU and HUU peers. Additionally, we hypothesized that HEU children are at a higher risk of oral diseases than HUU children. Finally, we hypothesized that oral health disparities exist among children living in urban and rural areas.

## Methods

### Study design and population

**Figure 1.**
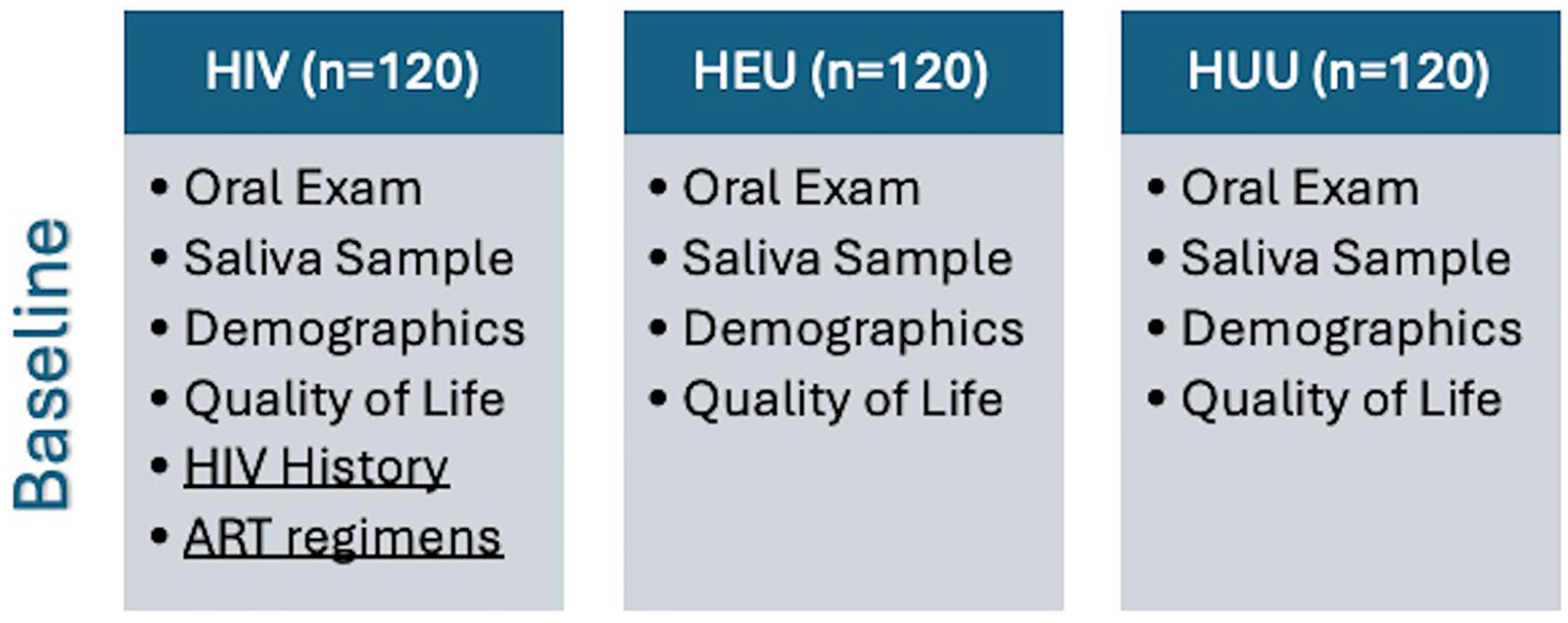
Variables collection at baseline for three cohorts HIV, HEU and HUU.

We recruited 360 children aged 3 years from 31 clinics at baseline from Kisumu County, Western Kenya, and stratified them by HIV status into three cohorts: CHIV (HIV, n = 120), HIV-exposed uninfected (HEU, n = 120), and HIV-unexposed uninfected (HUU, n = 120) (Malone, 2024). This article focuses only on the baseline characteristics of all enrolled children, as shown in Figure 1. While HIV+ children were recruited from the HIV treatment clinics, the HEU cohort were identified through the local Prevention of Mother to Child Transmission (PMTCT) clinics, and the HUU were recruited from a pool of patients receiving wellness checkup appointments at the Mother Child Health (MCH) clinics within the same hospital and/or community health clinic. All participants were recruited and enrolled between April 2023 and November 2023. Eligibility criteria included (1) being 3 years old at enrollment, (2) obtaining medical clearance from a primary care physician, (3) residing in Kisumu County for at least 12 months post-recruitment, and (4) being capable of providing saliva samples. Additionally, CHIV were required to have been on antiretroviral therapy (ART) for a minimum of 6 months. This study was conducted in accordance with the guidelines of the Declaration of Helsinki and approved by the Institutional Review Board (IRB) at the University of Washington (UW) (FWA #000006878) and Kenya’s Jaramogi Oginga Odinga Teaching and Referral Hospital Ethics Review Committee (ISERC/JOOTRH/688/23; Kenya). Written informed consent was obtained from all caregivers (parents and guardians), who were primarily responsible for the child, in their preferred language (English, Swahili, or Luo).

### Sample size and power calculation

Assuming a comparison of continuous measurements across three cohorts, with a pre-specified effect size of 0.3, a type I error rate of 0.05, and a desired power of at least 80%, we planned for three repeated measurements at baseline, 6 months, and 12 months. We assumed a high correlation of 0.8 between repeated measures (higher correlation values typically require larger sample sizes). Based on these parameters, a conservative sample size estimate of N = 100 per group (minimum N = 96) was required to detect an effect size of 0.3 with 80% power. To account for potential missing data and loss to follow-up, we recruited N = 120 children into each cohort at baseline.

### Measurements

Figure 1 presents the measures collected across the three cohorts. HIV history and ART regimens were collected only for CHIV. All children underwent detailed oral examinations, and saliva samples were collected at the time of the examination. Comprehensive demographic information was obtained at both baseline and study exit, along with assessments of the children’s oral health–related quality of life.

#### Dental examinations

Extraoral and intraoral clinical assessments of soft and hard tissues were done in accordance with the World Health Organization (WHO) Oral Health Surveys and Record Form for Oral Manifestations of HIV/AIDS (World Health Organization, 2013). With visual assessment augmented by headlamp, dental examinations were completed using disposable mirrors. A sequential examination of the teeth allowed for the identification of plaque accumulation, dental caries, dental trauma, dental fluorosis, missing teeth for other reasons, and other tooth-related pathologies.

Dental caries was assessed by presence (Y/N) and calculated in terms of the number of decayed teeth, missing teeth due to caries, and filled teeth/surfaces (dmft/dmfs for primary dentition). The dmft/dmfs index depicts previous and current dental disease and provides details on the severity of the caries in a child, as it reflects the number of teeth/tooth’s surfaces involved. For the evaluation of gingival health, tooth brushing was performed to determine gingival bleeding. All observations made were recorded in the oral health assessment form. Before the oral exam, the examiners were trained and calibrated by AK. The Kappa score for inter- and intra-calibration achieved 0.95 for both assessments.

#### Saliva samples and pH value

Due to the hot environmental temperatures in the study area, children were given water and a 30-minute waiting period, before saliva sample collection. This allowed for hydration and saliva composition normalization. Saliva was obtained using the passive drool technique to collect non-stimulated saliva. Children held their saliva for 15 seconds, then spit into a sterile tube during the oral evaluation. This process continued until the tube contained 5 mL of saliva or 5 minutes had passed. Salivary pH was measured using pH strips (Fisherbrand^™^ Plastic pH Strips; pH range 0–14). The specimen was then placed in a cooler bag, a photo of the pH strip was taken, and the readings were recorded in the participant’s oral health assessment form.

#### Demographics

Socio-demographic variables included the child’s age, sex, school type (public or private), and residence (rural, urban, or peri-urban). Health-related variables were extracted from the child’s medical records, and included HIV status (HIV, HEU, HUU).

#### HIV cohort

For CHIV children, additional details on viral load, other medications, caregiver-reported adherence, duration on antiretroviral treatment, information on initial and current ART regimens and dosages were obtained from medical records. HIV-related oral manifestations were also documented. Oral lesions were evaluated based on their location, type, and size, including any conditions observed, like candidiasis, salivary gland enlargement, and other common lesions.

### Statistical analysis

We compared the demographics among three cohorts, HIV, HEU, and HUU, using a one-way ANOVA for continuous variables, such as age, and chi-square test for categorical variables, such as sex, school type, and living location. We compared oral diseases and saliva pH among the three cohorts-HIV, HEU, and HUU-using chi-square tests among cohorts and one-way ANOVA in the same fashion. Logistic regression models were used to estimate the association between oral diseases and cohorts, adjusted for sex, school type, and living locations. All analyses were conducted using SAS 9.4. Statistical significance was determined at a p-value of less than 0.05.

## Results

### Cohort characteristics

A total of 360 Kenyan children aged 3 years participated in the study, with a mean age of 3.37 years (SD = 0.54), and 50.83% were female. Most children attended private school (47.22%), compared to public school (21.67%) or no school (31.11%). Most of the children lived in rural (48.60%), followed by urban (35.47%) and peri-urban (15.92%) areas. The cohorts did not significantly differ by age (p = 0.3501), sex (p = 0.7918), or location (p = 0.6973). However, school type varied significantly by HIV status (p = 0.0070), with most HUU children attending private school (Table 1). Each child in the HIV+ cohort (n = 120) was receiving ART treatment at the time of the study, with all current treatment regimens being nucleoside reverse transcriptase inhibitor (NRTI)-based. The overall duration of ART treatment ranged from 12 months or fewer to 49 months or more, with most children receiving treatment for 25–48 months (61.66%). The average duration of ART treatment was 29.89 months (SD = 14.38). Most children (65%) had a viral load of ≤50 copies/mL (considered undetectable), while 18.33% had viral loads between 50 and 400 copies/mL, and 16.67% had viral loads above 400 copies/mL. Most children (78.33%) achieved adequate ART adherence, defined as 90% or above.

### Oral disease prevalence by HIV status

The results of the dental exam (Table 2) showed 51% of children had active dental caries, with the highest prevalence in the HIV cohort (56.67%), compared to the HEU (43.33%) and HUU (53.33%) cohorts (p = 0.0990). Nearly every child had dental plaque (90.83%), and HIV cohort had the highest prevalence (93.33%), compared to HEU (89.17%) and HUU (90.00%) (p = 0.4963). Gingival bleeding upon brushing was observed in 40.83% of children, with the highest prevalence in the HIV cohort (45%), compared to the HEU (40.83%) and HUU (36.37%) (p = 0.4222). The HIV cohort had significantly higher levels of abnormal findings (80.83%), compared to the HEU (60.00%) and HUU (54.17%) (p < 0.0001). These abnormal findings included submandibular lymphadenopathy, parotid gland enlargement, geographic tongue, general skin rash, and perioral fungal infection. The distribution of enamel fluorosis, dental erosion, salivary gland swelling, angular cheilitis, herpetic lesions, and HPV/wart-like lesions could not be calculated due to the small number of cases per cohort. Intervention urgency for dental and oral findings was categorized into three groups: routine/preventative needs, non-urgent treatment needs, and urgent treatment needs (pain or infection). Most children fell into the routine/preventative needs category (72.23%), followed by non-urgent treatment needs (20%) and urgent treatment needs (7.78%) (p = 0.9075). Although the mean dmft score (3.4) and mean dmfs score (5.9) were both higher in the HIV cohort, the differences were not statistically significant (p = 0.0837, p = 0.2434, respectively). There was no significant difference in salivary pH levels among the three groups, with a mean pH of 6.7 (SD = 0.8) (p = 0.5086).

### Oral disease prevalence by HIV status and by residence

**Figure 2.**
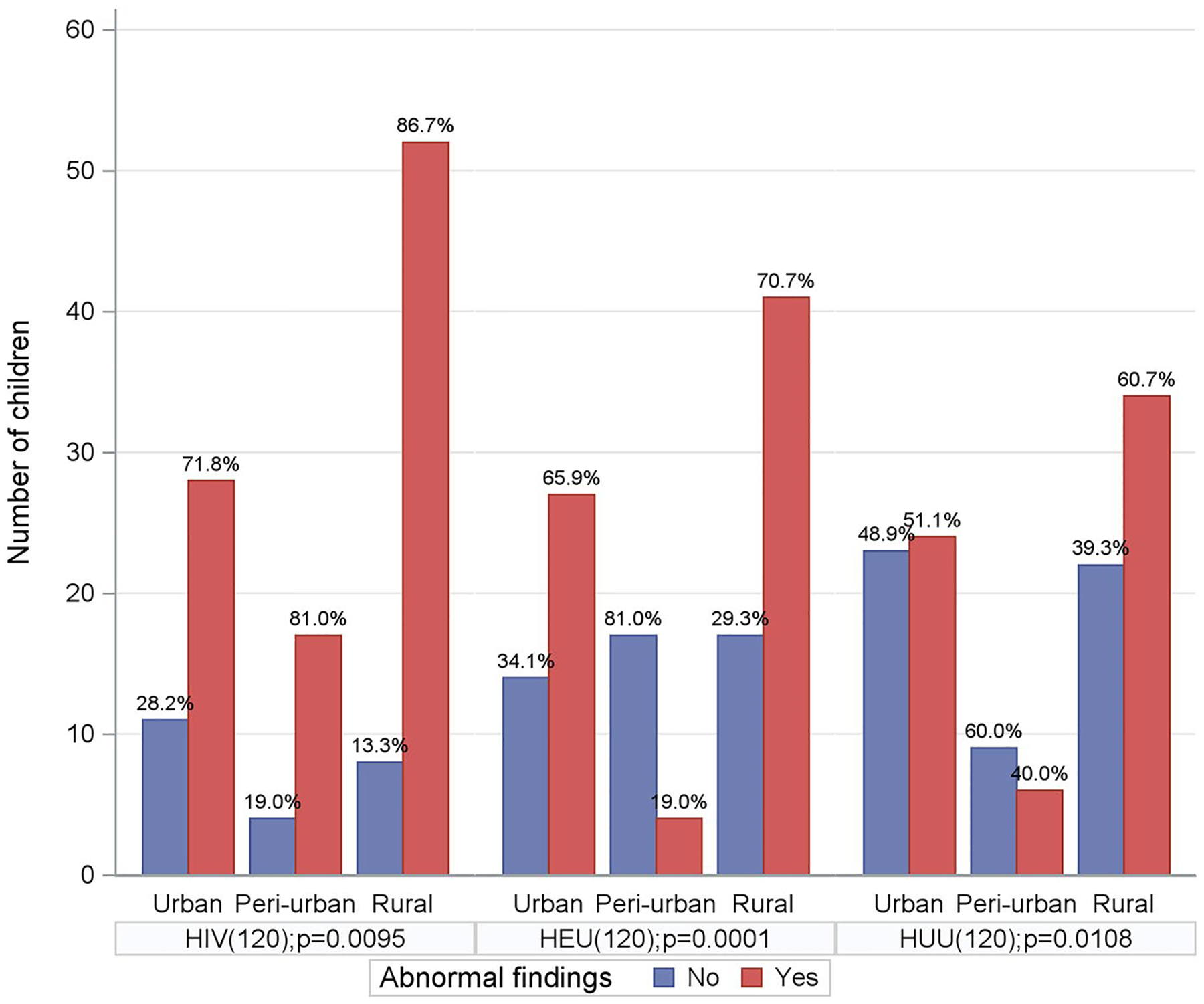
Abnormal findings by residence and by cohort.

Figure 2 presents the number and percentage of children with abnormal findings (Yes: red bars) and without abnormal findings (No: blue bars) across different residential settings (Urban, Peri-urban, and Rural) and HIV status cohorts. The length of each bar represents the number of children, and the numbers on the bars indicate the percentage of abnormal findings among children living in each residential area, by cohort group. Abnormal findings included submandibular lymphadenopathy, parotid gland enlargement, geographic tongue, general skin rash or perioral fungal infection. In each cohort, the prevalence of abnormal findings varied significantly by residential location. CHIV children had the highest prevalence of abnormal findings across all locations, with rural CHIV showing the highest prevalence (p = 0.0095). Among HEU children, those living in rural and urban areas had significantly higher prevalence of abnormal findings than their peri-urban counterparts (p = 0.0001). Among HUU children, those in rural areas had a higher prevalence of abnormal findings (p = 0.0108), while differences between urban and peri-urban areas were less pronounced. Overall, the figure highlights geographic disparities, with rural children across all cohorts experiencing the highest proportion of abnormal findings. The findings suggest a strong association between HIV status, residential location, and abnormal oral health outcomes.

### Association between characteristics of children and oral findings

Table 3 presents the results from the logistic regression analysis examining the association between four oral diseases, HIV status, and residential location, adjusted for sex and school attendance. HIV-positive individuals had significantly higher odds of abnormal findings (OR = 3.8, 95% CI: 2.09–6.92, p < 0.0001) and all combined conditions (OR = 3.96, 95% CI: 1.9–8.23, p = 0.0002). HEU individuals did not show significant differences in oral health conditions compared to HUU individuals. Regarding residence, rural residents had lower odds of active dental caries (OR = 0.46, 95% CI: 0.25–0.85, p = 0.0129) but significantly higher odds of abnormal findings (OR = 3.29, 95% CI: 1.74–6.25, p = 0.0003) and all combined conditions (OR = 2.98, 95% CI: 1.49–5.94, p = 0.002). These results highlight significant associations between HIV status, residential location, and oral health outcomes.

## Discussion

This article focuses on analyzing the baseline results of an ongoing longitudinal study aimed at understanding the impact of HIV infection and exposure on oral health among children in Western Kenya. We recruited children from the same area to minimize environmental factors impacting the three cohorts, reducing their potential contribution to differences in their oral health status and maximizing comparability among the three cohorts. Our findings indicate that CHIV have a significantly higher prevalence of abnormal oral findings (80.83%) compared to HEU (60%) and HUU children (54.17%) (p < 0.0001). These abnormal findings include submandibular lymphadenopathy, parotid gland enlargement, geographic tongue, generalized skin rash, and perioral fungal infection, as suggested by the WHO Basic Oral Health Survey, which standardize measurements of oral diseases and conditions to ensure international comparability (World Health Organization, 2013; Yeung, 2014).

Significant oral health disparities exist among children based on their residential setting, with differences observed between urban, rural, and peri-urban areas. Even among medically well-controlled CHIV, our results demonstrate that young children living with HIV have significantly higher levels of oral diseases compared to their HEU and HUU peers, with the highest burden observed in rural areas. We have confirmed disparities in the HIV epidemic and oral health among people living in urban, rural, and peri-urban areas (Kavanagh et al., 2021; Magadi, 2017). The rate of abnormal findings in each cohort shows that rural areas have a significantly higher prevalence than other areas. In the HIV cohort, children living in rural areas have the highest prevalence of oral diseases (86.7%). This highlights the urgent need for oral health care among this population (Okumu et al., 2022).

The uniqueness of our study lies in its focus on the 3-year-old in primary dentition stage among HIV-infected, HEU, and HUU children located in western Kenya. By recruiting children from the same county, we controlled for community-level confounding factors as well as age-related factors. The literature has consistently supported a high prevalence of dental caries in HIV-infected children compared to the general population over the past decades (Akhigbe et al., 2022; Coker et al., 2018; Hicks et al., 2000; Kikuchi et al., 2021; Wang et al., 2023). Specifically, a meta-analysis published in 2015, which examined dental caries rates in HIV-infected children and adolescents, reported an almost 200% increased odds of having dental caries in primary dentition compared to permanent dentition (OR: 2.98, 95% CI: 1.59–5.59, p = 0.0006) (Oliveira et al., 2015). The overall average dental caries prevalence for the 3–4-year-old children in our study was 51.1%, which was higher when compared to the general Kenyan pediatric population reported by the latest Kenya Oral Health Survey, where the general pediatric population (5-, 12-, and 15-year-olds) reported an average dental caries prevalence of 23.9% with the 5-year-old sub-population having the highest prevalence of 46.3% prevalence (Ministry of Health, 2022). Although there was no significant difference in dental caries prevalence amongst the three cohorts, the HIV cohort had the highest prevalence (56.7% compared to the HEU (43.33%) and HUU (53.33%)). Another study conducted in Nigeria found a significantly higher prevalence of dental caries, with an odds ratio of 2.6, among HIV-infected children compared to HUU children in the 0.5- to 6-year-old age group, but the difference was not significant among the HEU group (Akhigbe et al., 2022; Coker et al., 2018). Another study conducted in Mozambique in 2012 among children aged 1.7 to 16 years confirmed the finding that candidiasis was the most frequent oral lesion. Parotid enlargement was observed in 23% of cases, and the occurrence of mucosal lesions was higher in children who did not receive ART (Sales-Peres et al., 2012). We have previously found that the overall prevalence of oral diseases was higher among CHIV children (42%) compared to HEU (27%) and HUU (17%). The most reported conditions were candidiasis and oral HSV ulcers (Seminario et al., 2021). Unlike this study, our population were older children and adolescents residing in Nairobi, capital of Kenya. While the use of ART can significantly reduce the prevalence of oral lesions among people living with HIV (Zoman et al., 2024), yet oral diseases remain significantly more prevalent compared to uninfected individuals. We established a longitudinal cohort consisting of three groups of children and conducted evaluations at baseline to assess whether differences in oral diseases prevalence can be observed at such young age like 3 years and which factors are highly associated with its occurrences. This assessment will help us to establish baseline measurements and monitor these children’s oral innate immune system as they grow.

Peri-urban children had a higher prevalence of dental caries compared to their urban counterparts, which may reflect increased access to medical clinics in these areas, but limited access to dental clinics. Of interesting note, the prevalence of dental caries in the HEU group was markedly lower than the HIV and HUU groups. It is known that pregnant women with HIV in Kenya have access to various additional resources to help reduce the risk of transmission to the child (Hankin et al., 2007; Mmeje et al., 2019; Ngumbau et al., 2024). Even if the child is deemed uninfected after birth, the access to health care resources continues. From a medical standpoint, including HEU children provides valuable knowledge on determining the impact of anti-viral medications that mothers may have been taking during pregnancy (Hankin et al., 2007). This increased access to care is most notably different when comparing to the HUU cohort. The decrease in prevalence of dental caries within the HEU cohort could be attributed to the increasing initiatives to provide resources, maternal health education, along with increased frequency of medical exams to HIV-infected mothers and subsequently their exposed and uninfected children (Tuthill et al., 2024). These initiatives and programs to reduce mother to child transmission (MTCT) are notable in regions with the highest rates HIV transmission, which includes Kenya (Tuthill et al., 2024). Of interesting note, our study examiners interacting with the families at the clinic sites learned that the children born to mothers with HIV, who successfully prevented transmission to their child, were referred to as ‘miracle babies’. These children made up the HEU cohort. It was reported by our field team that these children were always well cared for and presented to each appointment in a timely manner, with superb hygiene habits. While these observations are anecdotical, it is relevant to bring up that the mothers and families of the HEU cohort utilized their access to resources to support the best possible outcomes for their children which may also translate the positive oral health outcomes discovered in this study.

We found that living in peri-urban areas was associated with the lowest prevalence of abnormal findings compared to those living in urban and rural areas across all cohorts. These findings suggest that living in a peri-urban location may be a protective factor against abnormal oral findings and all dental conditions. The peri-urban region in developing countries is in the transition zone between urban and rural settings, typically located at the perimeter of an urban city (Hutchings et al., 2022). Traditionally in Kenya, these areas were often home to poorer families with strained resources and markedly reduced access to healthcare leading to higher rates of infectious diseases, malnutrition, and mortality (Ndakuya-Fitzgerald et al., 2021; Ngugi et al., 2017). Previous studies suggested that access to HIV treatment and subsequent adequate adherence and disease management in urban setting was significantly higher than in non-urban or rural settings (Siika et al., 2009). However, more recent literature has suggested that there has been improved access to care in the peri-urban location due to efforts to provide mother-infant pairs with resources and early intervention for children born to HIV-positive mothers (Finocchario-Kessler et al., 2014; Hatzenbuehler et al., 2023; Truong et al., 2021). This new evidence supports our findings that the HIV-infected pediatric population living in peri-urban areas may be receiving increased healthcare resources, contributing to improved oral outcomes.

Although our study reports promising levels of viral suppression and adherence percentages within our pediatric HIV cohort, we still see increased prevalence of caries and significantly more abnormal oral findings when compared to the HEU and HUU cohorts. This may be due to the use of highly active antiretroviral therapy (HAART) medications, which often contain high-sugar syrups. It is important to consider what other factors may be influencing the occurrence and progression of oral diseases among the HIV pediatric population. One of those factors is salivary antimicrobial peptides (AMPs) and its association with dental caries and other oral diseases. We have previously found that concentrations of cathelicidin LL-37, a salivary AMP, were significantly high in children and adolescents living with HIV on early ART initiation (Seminario et al., 2024). In another study conducted in Nairobi, Kenya, on the oral microbiome among children living with HIV, we found distinct bacterial profiles and combinations associated with active dental caries. This may provide further evidence that different living environments contribute to these differences (Wang et al., 2025). Longitudinal studies assessing salivary AMPs as well as oral microbiome and their impact on oral health of HIV children are needed. Historically, oral manifestations, particularly oral candidiasis, were used for early detection of treatment resistance and/or viral immunosuppression (Coogan et al., 2005). Now with increasing ART success and potential decreasing presence of pediatric HIV oral manifestations, we must continue to explore other screening tools that dentists and allied health professionals can use as early signs to initiate interventions when needed. In this study, we report the most abnormal findings to be lymphadenopathy. Future studies should further analyze if the presence of lymphadenopathy, parotid gland enlargement or other abnormal oral, head or neck findings can serve as reliable markers for immunosuppression in HIV-infected pediatric patients. Future efforts may help dentists and other health care professionals to more efficiently screen and subsequently intervene when explicit values like viral load and CD4 counts may not be accessible.

This study has several limitations to address. First, our study was cross-sectional, and we only focused on baseline assessments in this manuscript. However, all three cohorts of 360 children are being followed longitudinally, which will provide a robust understanding of the oral immune system in the context of HIV, HEU, and HUU. In this study, we extracted medical data directly from health records, which may not reflect the most recent information at baseline. Additionally, oral examinations were conducted in a non-dental clinic, so oral health providers relied on visual clinical findings without access to radiographs. Furthermore, we did not collect data on diet, which is a potential confounding factor. Yet, this was the first study in the region that allowed oral health to be nested in the delivery of medical care of these very busy facilities. Since all children are from western Kenya, we assume their dietary habits are similar across cohorts. Overall, this study provides a foundation for understanding the oral health status of children at the primary dentition stage and its association with both HIV infection and exposure, as well as residency in urban, rural, and peri-urban settings. The oral health disparities observed across the three cohorts of children in different areas will help inform targeted intervention strategies and treatment recommendations. Improving access to health care among CHIV in rural areas and integrating oral health services into HIV care for targeted populations, may help enhance early diagnosis and reduce abnormal oral findings.

## Figures and Tables

**Table 1.** Characteristics of study population.

Variables	Total	HIV	HEU	HUU	p-value
	Mean (SD)	Mean (SD)	Mean (SD)	Mean (SD)	
	360 (100%)	120 (100%)	120 (100%)	120 (100%)	
Age					
Years	3.37 (0.54)	3.38 (0.54)	3.42 (0.54)	3.32 (0.55)	0.3501
Sex					
Female	183 (50.83)	58 (48.33)	63 (52.50)	62 (51.67)	0.7918
School type					
Public	78 (21.67)	29 (24.17)	32 (26.67)	17 (14.17)	0.007
Private	170 (47.22)	46 (38.22)	52 (43.33)	72 (60.00)	
No school	112 (31.11)	45 (37.50)	36 (30.00)	31 (25.83)	
Location					
Urban	127 (35.47)	39 (32.50)	41 (34.17)	47 (39.83)	0.6973
Peri-urban	57 (15.92)	21 (17.50)	21 (17.50)	15 (12.71)	
Rural	174 (48.60)	60 (50.00)	58 (48.33)	56 (47.46)	
Duration of ART					
12 months or less		20 (16.67)			
13–24 months		18 (15.00)			
25–36 months		37 (30.83)			
37–48 months		37 (30.83)			
49 months or more		8 (6.67)			
Viral load (copies/mL)					
VL <50, not detectable		78 (65.00)			
VL 50–400		22 (18.33)			
VL 400+		20 (16.67)			
Adherence					
≥95%		17 (14.17)			
90–94%		77 (64.16)			
<90%		26 (21.67)			

* Mean viral load (copies/mL) unable to include due to incomplete reporting, only 49 patients out of 120 reported their raw values.

**Table 2.** Bivariate analysis of distribution of HIV status with oral diseases.

Characteristics	Total	HIV	HEU	HUU	p-value
	360	120	120	120	
		Mean (SD)	Mean (SD)	Mean (SD)	
Oral findings					
Dental caries	184 (51.11)	68 (56.67)	52 (43.33)	64 (53.33)	0.0990
Dental plaque	327 (90.83)	112 (93.33)	107 (89.17)	108 (90.00)	0.4963
Gingival bleeding at brushing	147 (40.83)	54 (45.00)	49 (40.83)	44 (36.67)	0.4222
Abnormal findings*	234 (65.00)	97 (80.83)	72 (60.00)	65 (54.17)	<0.0001
Enamel fluorosis		8	9	7	NA**
Dental erosion		2	1	0	NA**
Salivary gland swelling		3	2	0	NA**
Other oral diseases					
Angular Cheilitis		1	0	3	NA**
Herpetic lesions		1	0	0	NA**
HPV/wart-like lesions		1	0	0	NA**
Intervention Urgency					
Routine/preventative needs	260 (72.23)	87 (72.50)	89 (74.17)	84 (70.00)	0.9075
Non-urgent treatment needs	72 (20.00)	25 (20.83)	21 (17.50)	26 (21.67)	
Urgent treatment needs (due to pain or infection)	28 (7.78)	8 (6.67)	10 (8.33)	10 (8.33)	
Salivary pH	6.7 (0.8)	6.7 (0.9)	6.8 (0.6)	6.8 (0.8)	0.5086
dmft score	2.8 (4.3)	3.4 (4.7)	2.2 (3.7)	2.9 (4.4)	0.0837
dmfs score	5 (10.5)	5.9 (11.2)	3.8 (8.6)	5.4 (11.5)	0.2534

* Abnormal findings include: submandibular lymphadenopathy, parotid gland enlargement, geographic tongue, general skin rash, perioral fungal infection.

** Unable to calculate p-values due to too small of sample size.

**Table 3.** Association among characteristics of Kenyan children and oral findings using multivariate logistic regression.

Variables	Category	Active dental caries		Bleeding on brush		Abnormal findings*		All combined condition**	
		OR (95%CI)	p-value	OR (95%CI)	p-value	OR (95%CI)	p-value	OR (95%CI)	p-value
HIV status (ref: HUU)	HIV	1.13, (0.67,1.9)	0.6401	1.4, (0.83,2.36)	0.2081	3.8, (2.09,6.92)	<.0001	3.96, (1.9,8.23)	0.0002
	HEU	0.66, (0.39,1.1)	0.1095	1.19, (0.71,2.01)	0.5158	1.31, (0.78,2.22)	0.3128	1.28, (0.72,2.3)	0.4005
Sex (ref: Female)	Male	1.29, (0.85,1.96)	0.2375	1.13, (0.74,1.72)	0.5838	1.28, (0.81,2.03)	0.2832	1.21, (0.72,2.05)	0.4707
In school (ref: Yes)	No school	1.02, (0.65,1.62)	0.9217	1.01, (0.64,1.6)	0.9682	1.05, (0.64,1.74)	0.8387	1.11, (0.62,1.97)	0.7267
Residence (ref: Peri-Urban)	Urban	0.58, (0.31,1.11)	0.0985	1.01, (0.53,1.91)	0.9883	2.05, (1.06,3.95)	0.0323	2.29, (1.13,4.65)	0.0223
	Rural	0.46, (0.25,0.85)	0.0129	1.38, (0.75,2.53)	0.3061	3.29, (1.74,6.25)	0.0003	2.98, (1.49,5.94)	0.002

* Abnormal findings include submandibular lymphadenopathy, parotid gland enlargement, geographic tongue, general skin rash, perioral fungal infection.

** All conditions include bleeding on brushing, abnormal findings or salivary gland swelling.

## Data Availability

Data are available to share with specific objective(s) requested by researchers through emails to corresponding author.
